# Supplementary material for: Booster Doses of Anti COVID-19 Vaccines: An Overview of Implementation Policies among OECD and EU Countries
Source: Int J Environ Res Public Health. 2022 Jun 13;19(12):7233. doi: 10.3390/ijerph19127233 (PMC9222878; doi:10.3390/ijerph19127233)
Supplement: Supplementary file 1 [file ijerph-19-07233-s001.zip › Supplementary material S1.pdf]

Supplementary material 1: Supplementary tables

Table S1. Clarification on definitions of terms used in the paper

**Term**	**Definition**
Starting date	Precise date of beginning of the booster campaign if declared in a state-level document, or month if not specifically indicated
Eligibility criteria	Requirements that must be met for a person to be included in the booster vaccination campaign
General population	All individuals without reference to any specific characteristic (age, clinical, work status, residency, etc.)
HCWs (healthcare workers)	Anyone who works in a healthcare or social care setting. It is a heterogeneous group including frontline healthcare workers and other healthcare workers; some also considered those not in direct contact with the patient
LTCF-Rs (long term care facilities residents)	People of any age resident in long term care facilities including nursing homes, rehabilitation facilities, inpatient behavioral health facilities, and long-term chronic care hospitals
VHRGs (vulnerable and high risk groups)	Generic definition that comprehends a heterogeneous clinical scenario (that could vary depending on the country of reference) such as a variety of health conditions like lung or heart disease, diabetes or conditions that affect the immune system
Authorized vaccines	Anti COVID-19 vaccines authorized or approved by each national regulatory agency and used to administer the booster dose
mRNA vaccines	COVID-19 vaccines approved for the vaccination campaign that use messenger ribonucleic acid (mRNA) molecules that contain instructions for the cells of the vaccinated person to synthesize viral Spike proteins, like BioNTech/Pfizer's Comirnaty or Moderna’s Spikevax
Interval	Recommended time interval between the primary vaccination course and the booster dose
Dosage	Amount of product administered for a single shot

Table S2. Products used for the COVID-19 booster dose, and policies by country.

	Vaccine			
**Country**	**Comirnaty**	**Spikevax**	**Vaxzevria**	**Janssen**
**Australia**	Initial recommendation: ≥18 yo, HCWs, VHRG, LTCF-R.<br><br>Dosage: 30 µg.	Not used	Not preferred. It can be used in case of:<br>• Individuals who have received Vaxzevria for their first two doses (if there are no contraindications or precautions for use)<br>• Significant adverse reaction occurred after a previous mRNA vaccine dose (reaction that contraindicates further doses: e.g., anaphylaxis, myocarditis).<br><br>Dosage: 0,5 ml.	Not used
**Belgium**	Initial recommendation: general population ≥ 65yo, HCWs, VHRG ≥ 12 yo, LTCF-R.<br><br>Later recommendation: general population ≥ 18 yo with preferred administration to people < 30 yo.<br><br>Dosage: 30 µg.	Initial recommendation: general population ≥ 65yo, HCWs, VHRG ≥ 12 yo, LTCF-R.<br><br>Later recommendation: general population ≥ 18 yo with preferred administration to people ≥ 30 yo.<br><br>Dosage: 50 µg.	Not used	Not used
**Canada**	Initial recommendation: general population ≥ 80 yo; ≥70 yo if completed the primary series with AZ, Covidshield or JJ; people in or from First Nations, Inuit and Métis; HCWs, LTCF-R.<br><br>Later recommendation: VHRG; general population gradually extended to ≥ 18 yo.<br><br>Exclusive use for < 30 yo.<br><br>Dosage: 30 µg.	Initial recommendation: general population ≥ 80 yo; ≥70 yo if completed the primary series with AZ, Covidshield or JJ; people in or from First Nations, Inuit and Métis.<br><br>Later recommendation: VHRG; general population gradually extended to ≥ 30 yo.<br><br>Dosage: 100 µg for LTCF residents, ≥ 70 yo and immunocompromised patients; 50 µg for other adults.	Last choice vaccine, off-label use only when other authorized COVID-19 vaccines are contraindicated or inaccessible	Last choice vaccine, off-label use only when other authorized COVID-19 vaccines are contraindicated or inaccessible
**Chile**	Initial recommendation: VHRG ≥ 16 yo, HCWs	Not used	Initial recommendation:	Not used

	and LTCF-R (prioritizing elderly).<br><br>Later recommendations: general population ≥ 45 yo (prioritizing elderly). Initially Pfizer was exclusively administered to immunocompromised patients, later only product available in the country as booster to all patients.<br><br>Dosage: 30 μg.		general population ≥ 55 yo and previously vaccinated with Coronavac.<br><br>Later recommendation: not used. A t the beginning Spikevax was temporarily offered to general population and later in the campaign was replaced by exclusive use of Comirnaty in all eligible patients.<br><br>Dosage: 0,5 ml.	
**Costa Rica**	Initial recommendation: not used.<br><br>Later recommendations: general population ≥ 65 yo, LTCF-R, VHRG.	Not used	Initial recommendation: first vaccine used as booster dose for HCWs, VHRG ≥ 18 yo.<br><br>Later recommendation: exclusive use for the booster shot in people aged 18-57 yo.	Not used
**Czech Republic**	Initial recommendation: ≥ 60 yo, VHRG, HCWs, social workers.<br><br>Preferably administered to individuals who received the same vaccine before.	Initial recommendation: ≥ 60 yo, VHRG, HCWs, social workers.<br><br>Preferably administered to individuals who received the same vaccine before.	Not used	Not used
**Cyprus**	Initial recommendation: general population ≥ 65 yo, VHRG , LTCF-R. Almost at the beginning of the campaign Cyprus stated that all adult population may be eligible.<br><br>Later recommendation: gradually including general population ≥ 30 yo and HCWs.<br><br>Recommended preferably for individuals who received Pfizer in the primary series, but also used for individuals	Initial recommendation: general population ≥ 65 yo, VHRG , LTCF-R. Almost at the beginning of the campaign Cyprus stated that all adult population may be eligible.<br><br>Later recommendation: gradually including general population ≥ 30 yo and HCWs.<br><br>Recommended preferably for individuals who received Spikevax in the primary series, but also used for	Not used	Initial recommendation: introduced later in the campaign compared to mRNA vaccines. Recommended to individuals who received Janssen first shot and that are ≥ 60 yo and/or HCWs and/or VHRG and/or LTCF-R.<br><br>Later recommendation: gradually including ≥30 yo general population.

	who received Spikevax, Vaxzevria or Janssen.	individuals who received Comirnaty, Vaxzevria or Janssen.		Exclusively used for those who received JJ in the primary series.
**Denmark**	Initial recommendation: general population ≥ 85 yo and/or individuals who received JJ in the primary series.<br><br>Later recommendation: general population >18 yo, HCWs, VHRG, LTCF-R.	Initial recommendation: general population ≥ 85 yo and/or individuals who received JJ in the primary series.<br><br>Later extended recommendation: general population >18 yo, HCWs, VHRG, LTCF residents.	Not used	Not used
**Estonia**	Initial recommendation: general population ≥ 65 yo, HCWs, VHRG, LTCF-R.<br><br>Later recommendation: general population ≥ 18 yo.<br><br>Dosage: 30 μg.	Initial recommendation: general population ≥ 65 yo, HCWs, VHRG, LTCF-R.<br><br>Later recommendation: general population ≥ 18 yo.<br><br>Dosage: 50 μg	Not used	Not used
**Finland**	Initial recommendation: general population ≥ 60 yo, HCWs, VHRG, LTCF-R and persons who have been vaccinated at a short vaccination interval of less than 6 weeks (first series).	Initial recommendation: general population ≥ 60 yo, HCWs, VHRG, LTCF-R and persons who have been vaccinated at a short vaccination interval of less than 6 weeks (first series) .	Not used	Not used
**France**	Initial recommendation: general population ≥ 65 yo, VHRG ≥ 12 yo, HCWs and LTCF-R.<br><br>Later recommendation: approved for ≥ 18 aa, exclusive use of Pfizer ≤ 30 yo.<br><br>Dosage 30 μg	Initial recommendation: general population ≥ 65 yo, VHRG ≥ 12 yo, HCW and LTCF-R.<br><br>Later recommendation: lowered the cut off to > 30 yo.<br><br>Dosage 50 μg	Not used	Not used
**Germany**	Initial recommendation: general population ≥ 18 yo (priority to VHRG and ≥ 70 yo), HCWs, VHRG and LTCF-R.<br><br>Exclusive use of Pfizer in < 30 yo.	Initial recommendation: general population ≥ 30 yo (priority to VHRG and ≥ 70 yo), HCWs, VHRG and LTCF-R.	Not used	Not used
**Iceland**	Initial recommendation: individuals ≥ 16 yo with a history of COVID-19, individuals without history of COVID-19 antibodies who were vaccinated with the Janssen vaccine, LTCF-	Initial recommendation: individuals ≥ 16 yo with a history of COVID-19, individuals without history of COVID-19 antibodies who were vaccinated with the Janssen vaccine, LTCF-	Not used	Not used

	R ≥ 60 yo, VHRG, general population ≥ 60 yo.<br><br>Later recommendation: individuals 12-17 yo with a history of COVID-19, general population ≥ 16 yo. Exclusive use of Pfizer in 16-39 yo.<br><br>Dosage 30 µg	R ≥ 60 yo, VHRG, general population ≥ 60 yo.<br><br>Later recommendation: individuals 12-17 yo with a history of COVID-19, general population ≥ 40 yo.<br><br>Dosage 50 µg		
**Ireland**	Initial recommendation: general population ≥ 60 yo (at the beginning of the campaign already stated the priority groups up to and including ≥ 16 yo), VHRG ≥ 16 yo, HCWs and LTCF-R.<br><br>Exclusive use of Pfizer in ≤ 30 yo.<br><br>Dosage 30 µg	Initial recommendation: general population ≥ 60 yo (although already approved and stated the priority groups up to and including ≥ 16 yo), VHRG ≥ 30 yo, HCWs and LTCF-R.<br><br>Approved as booster in > 30 yo.<br><br>Dosage 50 µg	Not used	Not used
**Israel**	Initial recommendation: general population ≥12 yo (prioritizing seniors and starting from ≥ 60 yo).<br><br>Those who received Pfizer as the first vaccine series shall be revaccinated with the same vaccine.	Initial recommendation: to ≥ 18 yo (prioritizing seniors and starting from ≥ 60 yo).	Not used	Not used
**Italy**	Initial recommendation: general population ≥ 80 yo, HCWs ≥ 60 yo, LTCF-R and VHRG ≥ 18 yo.<br><br>Later recommendation: general population ≥ 18 yo.<br><br>Dosage 30 µg	Initial recommendation: general population ≥ 80 yo, HCWs ≥ 60 yo, LTCF-R and VHRG ≥ 18 yo.<br><br>Later recommendation: general population ≥ 18 yo.<br><br>Dosage 50 µg	Not used	Not used
**Luxembourg**	Initial recommendation: general population ≥ 75 yo; VHRG and LTCF residents without any age limit.<br><br>Later recommendation: lowered the age cut-off up to and including ≥ 65 yo, HCWs excluding however VHRG.	Initial recommendation: general population ≥ 75 yo; VHRG and LTCF residents without any age limit.<br><br>Later recommendation: lowered the age cut-off up to and including ≥ 65 yo, HCWs excluding however VHRG.	Not used	Not used

	Preferably same product of the first vaccination series.<br><br>Dosage 30 µg.	Preferably same product of the first vaccination series.<br><br>Dosage 50 µg.		
**Malta**	Initial recommendation: general population ≥ 12 yo (prioritizing seniors).	Initial recommendation: general population ≥ 12 yo (prioritizing seniors).	Not used	Not used
**Netherlands**	Initial recommendation: general population ≥ 80 yo.<br><br>Later recommendation: general population ≥ 18 yo (prioritizing seniors), HCWs (including social workers), VHRG, LTCF-R.<br><br>Dosage 30 µg	Initial recommendation: general population ≥ 80 yo.<br><br>Later recommendation: general population ≥ 18 yo (prioritizing seniors), HCWs (including social workers), VHRG, LTCF-R.<br><br>Dosage 50 µg	Not used	Not used
**New Zealand**	Initial recommendation: general population ≥18 yo, HCWs, VHRG, LTCF-R.<br><br>Dosage 30 µg	Not used	Initial recommendation: general population ≥18 yo, HCWs, VHRG, LTCF-R.<br><br>Dosage 0,5 ml	Not used
**Norway**	Initial recommendation: general population ≥ 65 yo (prioritizing elderly and stating that all adult population will receive the booster), LTCF-R, VHRG ≥ 18 yo<br><br>Later recommendation: general population ≥ 45 yo (prioritizing elderly) and HCWs.<br><br>Dosage 30 µg	Initial recommendation: general population ≥ 65 yo (prioritizing elderly and stating that all adult population will receive the booster), LTCF-R, VHRG ≥ 18 yo<br><br>Later recommendation: general population ≥ 45 yo (prioritizing elderly) and HCWs.<br><br>Dosage 30 µg	Not used	Not used
**Portugal**	Initial recommendation: general population ≥ 65 yo ("open house" access ≥ 80 yo), HCWs (including social workers and fire fighters involved in patients transport) LTCF-R.<br><br>Later recommendation: individuals 12-65 yo that received Janssen as first vaccine series.	Initial recommendation: general population ≥ 65 yo ("open house" access ≥ 80 yo), HCWs (including social workers and fire fighters involved in patients transport) LTCF-R.<br><br>Later recommendation: individuals 12-65 yo that received Janssen as first vaccine series.	Not used	Not used
**Republic of Korea**	Initial recommendation: general population ≥ 60 yo, HCWs, VHRG, LTCF-R (specifically facilities vulnerable for outbreak).	Initial recommendation: general population ≥ 60 yo, HCWs, VHRG, LTCF-R (specifically facilities vulnerable for outbreak).	Not used	Not used

**Spain**	Initial recommendation: general population ≥ 70 yo, VHRG, LTCF-R.<br><br>Later recommendation: general population ≥ 60, HCWs.<br><br>Dosage 30 μg	Initial recommendation: general population ≥ 70 yo, VHRG, LTCF-R.<br><br>Later recommendation: general population ≥ 60, HCWs.<br><br>Dosage 50 μg	Not used	Not used
**Sweden**	Initial recommendation: general population ≥ 80 yo, HCWs, VHRG and those in proximity (staff and relatives), LTCF-R.<br><br>Later recommendation: general population ≥ 18 yo (prioritizing elderly)<br><br>Dosage 30 μg	Initial recommendation: general population ≥ 80 yo, HCWs, VHRG and those in proximity (staff and relatives), LTCF-R.<br><br>Later recommendation: general population ≥ 18 yo (prioritizing elderly)<br><br>Dosage 50 μg	Not used	Not used
**Switzerland**	Initial recommendation: general population ≥ 65 yo and ≥ 16 yo if VHRG or LTCF residents.<br><br>Later recommendation: HCWs.<br><br>Dosage 30 μg	Initial recommendation: general population ≥ 65 yo and ≥ 16 yo if VHRG or LTCF residents.<br><br>Later recommendation: HCWs.<br><br>Dosage 50 μg	Not used	Not used
**UK**	Initial recommendation: general population ≥ 40 yo and approved from 18 yo and older. HCWs (including social workers), VHRG ≥ 16 yo.<br><br>Dosage 30 μg	Initial recommendation: general population ≥ 40 yo and approved from 18 yo and older. HCWs (including social workers), VHRG ≥ 16 yo.<br><br>Dosage 50 μg	May be considered when mRNA vaccines can not be offered due to contraindication.	Not used
**USA**	Initial recommendation: general population ≥ 65 yo, VHRG ≥ 50, LTCF-R and HCW ≥ 18 yo.<br><br>Later recommendation: general population ≥ 18 yo	Initial recommendation: general population ≥ 65 yo, VHRG ≥ 50, LTCF-R and HCW ≥ 18 yo.<br><br>Later recommendation: general population ≥ 18 yo	Not used	Initial recommendation: general population ≥ 65 yo, VHRG ≥ 50, LTCF-R and HCW ≥ 18 yo.<br><br>Later recommendation: general population ≥ 18 yo

LTCF-R =long term care facilities residents, HCWs= health care workers. * VHRG= "VHRG" is a generic definition that comprehend an heterogeneous clinical scenario that could vary depending on the country of reference. As for later recommendation, all "initial recommendation" are still valid in "later recommendation" unless specified otherwise.

Table S3. Time intervals between the primary vaccination series and the booster dose depending on the vaccine administered as booster by country.

	Vaccine			
**Country**	**Comirnaty**	**Spikevax**	**Vaxzevria**	**Janssen**
**Australia**	6 months interval for all individuals eligible regardless of the vaccine used for the primary series.	Not used	6 months interval for all individuals eligible regardless of the vaccine used for the primary series.	Not used
**Belgium**	Individuals who completed the primary series with an mRNA vaccine: 6 months.<br><br>Individuals who completed the primary series with AZ: 4 months.<br><br>Individuals who completed the primary series with JJ: 4 months; later reduced to 2 months.	Individuals who completed the primary series with an mRNA vaccine: 6 months.<br><br>Individuals who completed the primary series with AZ: 4 months.<br><br>Individuals who completed the primary series with JJ: 4 months; later reduced to 2 months.	Not used	Not used
**Canada**	6 months interval for all individuals eligible regardless of the vaccine used for the primary series.	6 months interval for all individuals eligible regardless of the vaccine used for the primary series.	6 months interval regardless of the vaccine used for the primary series.	Not used
**Chile**	Initial recommendation: 4 months for general population and 2 months for immunocompromised patients.<br><br>Later recommendation: 6 months for all individuals.	Not used	Initial recommendation: 4 months for general population.<br><br>Later recommendation: Not used, replaced by Comirnaty.	Not used
**Costa Rica**	Unknown	Not used	Unknown	Not used
**Czech Republic**	Initial recommendation: 6 months for all individuals regardless of the vaccine used for the primary series.<br><br>Later recommendation: 5 months for all individuals regardless of	Initial recommendation: 6 months for all individuals regardless of the vaccine used for the primary series.<br><br>Later recommendation: 5 months for all individuals regardless of	Not used	Not used

	the vaccine used for the primary series.	the vaccine used for the primary series.		
**Cyprus**	6 months interval for all individuals eligible regardless of the vaccine used for the primary series.	6 months interval for all individuals eligible regardless of the vaccine used for the primary series.	Not used	6 months
**Denmark**	6 months interval for all individuals eligible regardless of the vaccine used for the primary series.	6 months interval for all individuals eligible regardless of the vaccine used for the primary series.	Not used	Not used
**Estonia**	Individuals who completed the primary series with an mRNA vaccine: 6 months.<br><br>Individuals who completed the primary series with AZ or JJ: 5 months.	Individuals who completed the primary series with an mRNA vaccine: 6 months.<br><br>Individuals who completed the primary series with AZ or JJ: 5 months.	Not used	Not used
**Finland**	6 months interval for all individuals eligible regardless of the vaccine used for the primary series.	6 months interval for all individuals eligible regardless of the vaccine used for the primary series.	Not used	Not used
**France**	Initial recommendation: 6 months for all individuals eligible.<br><br>Later recommendation: 5 months for general population and 3 months for HRCD patients.	Initial recommendation: 6 months for all individuals eligible.<br><br>Later recommendation: 5 months for general population and 3 months for HRCD patients.	Not used	Not used
**Germany**	6 months interval for all individuals regardless of the vaccine used for the primary series. Moreover they generally stated that "In individual cases or if there is enough vaccine available, booster can be given after just five months"	6 months interval for all individuals regardless of the vaccine used for the primary series. Moreover they generally stated that "In individual cases or if there is enough vaccine available, booster can be given after just five months"	Not used	Not used
**Iceland**	Initial recommendation: individuals who completed the primary series with an mRNA vaccine: 5-6 months, individuals who	Initial recommendation: individuals who completed the primary series with an mRNA vaccine: 5-6 months, individuals who	Not used	Not used

	completed the primary series JJ: 4 weeks.<br><br>Later recommendation: 5-6 months interval for all individuals regardless of the vaccine used for the primary series.	completed the primary series JJ: 4 weeks.<br><br>Later recommendation: 5-6 months interval for all individuals regardless of the vaccine used for the primary series.		
**Ireland**	6 months interval for all individuals eligible regardless of the vaccine used for the primary series.	6 months interval for all individuals eligible regardless of the vaccine used for the primary series.	Not used	Not used
**Israel**	5 months interval for all individuals eligible regardless of the vaccine used for the primary series.	6 months interval for all individuals eligible regardless of the vaccine used for the primary series.	Not used	Not used
**Italy**	Initial recommendation: 6 months for all individuals eligible.<br><br>Later recommendation: 5 months for all individuals eligible.	Initial recommendation: 6 months for all individuals eligible.<br><br>Later recommendation: 5 months for all individuals eligible.	Not used	Not used
**Luxembourg**	6 months interval for general population eligible that completed the primary series with an mRNA vaccine, 2 months if primary series completed with Janssen.	6 months interval for general population eligible that completed the primary series with an mRNA vaccine, 2 months if primary series completed with Janssen.	Not used	Not used
**Malta**	3 months interval for all individuals eligible regardless of the vaccine used for the primary series.	3 months interval for all individuals eligible regardless of the vaccine used for the primary series.	Not used	Not used
**Netherlands**	6 months interval for all individuals eligible regardless of the vaccine used for the primary series.	6 months interval for all individuals eligible regardless of the vaccine used for the primary series.	Not used	Not used
**New Zealand**	6 months interval for all individuals eligible regardless of the vaccine used for the primary series.	Not used	6 months interval for all individuals eligible regardless of the vaccine used for the primary series.	Not used
**Norway**	Initial recommendation: 6 months for all	Initial recommendation: 6 months for all	Not used	Not used

	individuals eligible.<br><br>Later recommendation: 5 months for all individuals eligible.	individuals eligible.<br><br>Later recommendation: 5 months for all individuals eligible.		
**Portugal**	Individuals who completed the primary series with an mRNA vaccine: 5 months interval.<br><br>Individuals who completed the primary series with Janssen: 3 months interval.	Individuals who completed the primary series with an mRNA vaccine: 5 months interval.<br><br>Individuals who completed the primary series with Janssen: 3 months interval.	Not used	Not used
**Republic of Korea**	6 months interval for all individuals eligible regardless of the vaccine used for the primary series.	6 months interval for all individuals eligible regardless of the vaccine used for the primary series.	Not used	Not used
**Spain**	Initial recommendation: 6 months for all individuals eligible regardless of the product used in the primary series.<br><br>Later recommendation: 3 months for all individuals eligible who completed the primary series with AZ or JJ.	Initial recommendation: 6 months for all individuals eligible regardless of the product used in the primary series.<br><br>Later recommendation: 3 months for all individuals eligible who completed the primary series with AZ or JJ.	Not used	Not used
**Sweden**	6 months for general population, 5 months for patients > 65 yo	6 months for general population, 5 months for patients > 65 yo	Not used	Not used
**Switzerland**	6 months interval for all individuals eligible regardless of the vaccine used for the primary series.	6 months interval for all individuals eligible regardless of the vaccine used for the primary series.	Not used	Not used
**UK**	Initial recommendation: 6 months for all individuals eligible regardless of the product used in the primary series.<br><br>Later recommendation: 3 months for all individuals eligible regardless of the	Initial recommendation: 6 months for all individuals eligible regardless of the product used in the primary series.<br><br>Later recommendation: 3 months for all individuals eligible regardless of the	6 months in those cases where mRNA vaccines can not be used due to contraindications.	Not used

	product used in the primary series.	product used in the primary series.		
**USA**	Individuals who completed the primary series with an mRNA vaccine: 6 months interval.<br><br>Individuals who completed the primary series with Janssen: 2 months interval.	Individuals who completed the primary series with an mRNA vaccine: 6 months interval.<br><br>Individuals who completed the primary series with Janssen: 2 months interval.	Not used	Individuals who completed the primary series with an mRNA vaccine: 6 months interval.<br><br>Individuals who completed the primary series with Janssen: 2 months interval.
