# Supplementary material for: Booster Doses of Anti COVID-19 Vaccines: An Overview of Implementation Policies among OECD and EU Countries
Source: Int J Environ Res Public Health. 2022 Jun 13;19(12):7233. doi: 10.3390/ijerph19127233 (PMC9222878; doi:10.3390/ijerph19127233)
Supplement: Supplementary file 1 [file ijerph-19-07233-s001.zip › Supplementary material S2.pdf]

Supplementary material 2: List of included documents

Due to the rapid updates of indications and guidelines, some sources may not currently be reachable on the web. Screenshots of all the web pages used for the drafting of the article are available from the corresponding author on reasonable request.

Table of contents

AUSTRALIA ... 2

BELGIUM ... 2

CANADA ... 3

CHILE ... 4

COSTARICA ... 5

CZECH REPUBLIC ... 5

CYPRUS ... 6

DENMARK ... 7

ESTONIA ... 7

FINLAND ... 8

FRANCE ... 8

GERMANY ... 9

ICELAND ... 9

IRELAND ... 9

ISRAEL ... 10

ITALY ... 11

LUXEMBOURG ... 12

MALTA ... 13

NETHERLANDS ... 13

NEW ZEALAND ... 14

NORWAY ... 15

PORTUGAL ... 15

REPUBLIC OF KOREA ... 16

SPAIN ... 16

SWEDEN ... 17

SWITZERLAND ... 17

UNITED KINGDOM ... 18

UNITED STATES OF AMERICA ... 18

AUSTRALIA

From: https://www.health.gov.au/; acquired: 07/12/2021:

- Australian Technical Advisory Group on Immunisation (ATAGI) recommendations on the use of a booster dose of COVID-19 vaccine, 27 October 2021
- Recommendations on the use of a 3rd primary dose of COVID-19 vaccine in individuals who are severely Immunocompromised, Version 1.1. 2 November 2021
- Australian Technical Advisory Group on Immunisation (ATAGI) Clinical guidance on use of COVID-19 vaccine in Australia, Version 7.4 29 October 2021

Other sources available from:

- Australian Government, Department of Health; "Australian Health Protection Principal Committee (AHPPC) statement on mandatory vaccination of aged care in-home and community aged care workers" https://www.health.gov.au/news/australian-health-protection-principal-committee-ahppc-statement-on-mandatory-vaccination-of-aged-care-in-home-and-community-aged-care-workers; acquired: 07/12/2021
- Act Government, https://www.covid19.act.gov.au/stay-safe-and-healthy/vaccine/covid-19-vaccines-third-doses-and-booster-doses#COVID-19-vaccine-booster-doses-for-the-general-population; acquired: 07/12/2021

BELGIUM

From: Federal Public Service (FPS) Health, Food Chain Safety and Environment; https://www.health.belgium.be/fr; acquired: 09/12/2021:

- CO-ADMINISTRATION DES VACCINS CONTRE LA COVID-19 AVEC D'AUTRES VACCINS (VACCINATION SIMULTANÉE), OCTOBRE 2021, CSS N° 9675
- A BOOSTER DOSE OF mRNA COVID-19 VACCINATION FOR HEALTHCARE WORKERS NOVEMBER 2021, SHC No 9679
- VACCINATION CONTRE LE COVID-19 POUR LE PERSONNEL DE SANTÉ : BESOIN D'UN ENCADREMENT SUPPLÉMENTAIRE EN MATIÈRE D'OBLIGATION ?, NOVEMBRE 2021, CSS N° 9671
- BOOSTER VACCINATION AGAINST COVID-19 FOR THE GENERAL POPULATION, NOVEMBER 2021, SHC No 9683

- BOOSTER DOSE OF AN mRNA VACCINE FOR INDIVIDUALS VACCINATED AGAINST COVID-19 WITH THE JANSSEN VACCINE, DECEMBER 2021, SHC No 9677
- VACCINATION POUR LES PERSONNES ÂGÉES, IMMUNODÉPRIMÉES ET MALADES CHRONIQUES : UNE DOSE SUPPLÉMENTAIRE DE VACCIN ARNM CONTRE LA COVID-19 PARTIE 1 : PERSONNES ÂGÉES, SEPTEMBRE 2021, CSS N° 9650

Other sources available from:

- Federal Public Service of Publi Health; "Covid safe ticket"; " Safely through the winter"; https://www.info-coronavirus.be/fr/; acquired: 09/12/2021
- Coronavirus Brussels | Site Officiel de la Région Bruxelloise; "Vaccination de rappel contre le Covid-19 pour les personnes immunodéprimées"; "Où se faire vacciner à Bruxelles"; https://coronavirus.brussels/en/home-2/; acquired: 09/12/2021

CANADA

From: https://www.canada.ca/en/public-health/services/immunization/national-advisory-committee-on-immunization-naci.html; Acquired: from 16/11/2021 to 06/12/2021:

- SUMMARY OF NATIONAL ADVISORY COMMITTEE ON IMMUNIZATION (NACI) STATEMENT OF OCTOBER 29, 2021
- SUMMARY OF THE NATIONAL ADVISORY COMMITTEE ONIMMUNIZATION (NACI) STATEMENT OF DECEMBER 3, 2021, Guidance on booster COVID-19 vaccine doses in Canada – Update December 3, 2021
- An Advisory Committee Statement (ACS) National Advisory Committee on Immunization (NACI) Guidance on booster COVID-19 vaccine doses in Canada – Update December 3, 2021

Other sources available from:

Government of Canada; Guidance on booster COVID-19 vaccine doses in Canada; https://www.canada.ca/en/public-health/services/immunization/national-advisory-committee-on-immunization-naci/reccomendations-use-covid-19-vaccines/statement-guidance-booster-doses.html; acquired: 06/12/2021

CHILE

From: Ministerio de Salud; https://www.minsal.cl/; Acquired: 16/12/2021:

- DOSIS DE REFUERZO DE VACUNA CONTRA SARS-COV-2 EN PACIENTES INMUNOCOMPROMETIDOS SUBSECRETARIA DE SALUD PÚBLICA DIVISIÓN DE PREVENCIÓN Y CONTROL DE ENFERMEDADES DEPARTAMENTO DE INMUNIZACIONES, 19 de julio 2021
- DOSIS DE REFUERZO EN LA CAMPAÑA DE VACUNACIÓN CONTRA SARS-COV-2 EN CHILE DEPARTAMENTO DE INMUNIZACIONES DIVISIÓN DE PREVENCIÓN Y CONTROL DE ENFERMEDADES SUBSECRETARIA DE SALUD PÚBLICA MINISTERIO DE SALUD, 05 de agosto 2021
- DOSIS DE REFUERZO EN LA CAMPAÑA DE VACUNACIÓN CONTRA SARS-COV-2 EN CHILE DEPARTAMENTO DE INMUNIZACIONES DIVISIÓN DE PREVENCIÓN Y CONTROL DE ENFERMEDADES SUBSECRETARIA DE SALUD PÚBLICA, MINISTERIO DE SALUD, Actualización al 16 de agosto 2021
- DOSIS DE REFUERZO EN LA CAMPAÑA DE VACUNACIÓN CONTRA SARS-COV-2 EN CHILE, 05 de octubre 2021, DEPARTAMENTO DE INMUNIZACIONES DIVISIÓN DE PREVENCIÓN Y CONTROL DE ENFERMEDADES SUBSECRETARIA DE SALUD PÚBLICA MINISTERIO DE SALUD
- ACTUALIZACIÓN AL PLAN, Paso a Paso nos cuidamos, Válido desde el 1 de noviembre de 2021

Other sources available from:

- Ministerio de Salud; "Calendario de vacunación masiva contra COVID-19"; https://www.minsal.cl/calendario-de-vacunacion-masiva-contra-covid-19/; acquired: 16/12/2021
- Sociedad Chilena de Infectología; "Recomendación del CAVEI sobre la introducción de una dosis de refuerzo al plan de vacunación COVID-19"; https://sochinf.cl/recomendacion-del-cavei-sobre-la-introduccion-de-una-dosis-de-refuerzo-al-plan-de-vacunacion-covid-19/; acquired: 15/12/2021

COSTARICA

– Ministerio de Salud Costa Rica; "Población inmunosuprimida también recibirá tercera dosis contra COVID-19 en el 2022"; https://www.ministeriodesalud.go.cr/index.php/prensa/43-noticias-2021/1093-poblacion-inmunosuprimida-tambien-recibira-tercera-dosis-contra-covid-19-en-el-2022; acquired: 16/12/2021

– Ministerio de Salud Costa Rica; "Tercera dosis COVID-19 comenzará a aplicarse en centros de larga estancia"; https://www.ministeriodesalud.go.cr/index.php/prensa/43-noticias-2021/1171-tercera-dosis-covid-19-comenzara-a-aplicarse-en-centros-de-larga-estancia; acquired: 16/12/2021

– Ministerio de Salud Costa Rica; "Terceras dosis COVID-19 son para personal de primera respuesta y hogares de larga estancia, resto de población recibe tercera dosis solo como plan de contingencia"; https://www.ministeriodesalud.go.cr/index.php/prensa/43-noticias-2021/1175-terceras-dosis-covid-19-son-para-personal-de-primera-respuesta-y-hogares-de-larga-estancia-resto-de-poblacion-recibe-tercera-dosis-solo-como-plan-de-contingencia; acquired: 16/12/2021

– Ministerio de Salud Costa Rica; "A partir del 14 de diciembre inicia apertura de la vacunación de terceras dosis contra COVID-19 en mayores de 65 años"; https://www.ministeriodesalud.go.cr/index.php/prensa/43-noticias-2021/1178-a-partir-del-14-de-diciembre-inicia-apertura-de-la-vacunacion-de-terceras-dosis-contra-covid-19-en-mayores-de-65-anos; acquired: 16/12/2021

– Presidencia; "CCSS INICIA APLICACIÓN DE TERCERA DOSIS A PERSONAL DE PRIMERA RESPUESTA"; https://www.presidencia.go.cr/comunicados/2021/10/ccss-inicia-aplicacion-de-tercera-dosis-a-personal-de-primera-respuesta/; acquired: 16/12/2021

CZECH REPUBLIC

– Ministry of Health of the Czech Republic; "Booster dose"; https://covid.gov.cz/en/situations/register-vaccination/booster-and-additional-dose; acquired: 03/12/2021

– Ministry of Health of the Czech Republic; "Vaccination timeline"; https://covid.gov.cz/en/situations/register-vaccination/vaccination-timeline; acquired: 03/12/2021

– Ministry of Health of the Czech Republic; "Central covid-19 vaccination booking system"; https://registrace.mzcr.cz/; acquired: 03/12/2021

CYPRUS

From: https://www.moh.gov.cy/moh/moh.nsf/index_en/index_en; acquired: 06/12/2021:

– REPUBLIC OF CYPRUS MINISTRY OF HEALTH Press release 2 September 2021, "ECDC and EMA highlight considerations for additional and booster doses of COVID-19 vaccines"

– PRESS RELEASE PRESS AND INFORMATION OFFICE (P.I.O.) 15.10.2021 "Council of Ministers: The age limit for the administration of the booster/3rd dose is reduced to 60 and new vulnerable groups are added"

– "Extension of measures against the spread of COVID-19 — A booster dose available for individuals vaccinated with J&J", 27.10.2021

– "Extension of measures against the spread of COVID-19 — A booster dose available for people over 50 and for individuals with severe obesity", 4.11.2021

– "Booster dose administration procedure for people who were vaccinated with the Johnson&Johnson, for diabetics and for people aged 50 and over", 5.11.2021

– "Administration of a booster dose for individuals aged 40 years and over, begins", 16.11.2021

– "Administration of the booster/3rd dose of the vaccine against COVID-19 for individuals aged 30 and over", 29.11.2021

Other sources available from:

– Republic of Cyprus Ministry of Health; "Vaccination Portal opens for 3rd dose vaccination appointments"; https://www.pio.gov.cy/coronavirus/uploads/02102021_vaccinationover80yoEN.pdf; acquired: 06/12/2021

– Press and Information Office, Ministry of Interior, Republic of Cyprus; "The Ministry of Health proceeds with the administration of the 3rd dose of the COVID-19 vaccine to persons 65 years of age and older"; https://www.pio.gov.cy/en/press-releases-article.html?id=23305#flat; acquired: 06/12/2021

DENMARK

- Danish Health Authority; “Target groups for vaccination”; https://www.sst.dk/en/English/Corona-eng/Vaccination-against-COVID-19/Who-should-be-vaccinated; acquired: 06/12/2021
- Danish Health Authority; “How you will get vaccinated”; https://www.sst.dk/en/English/Corona-eng/Vaccination-against-COVID-19/How-will-you-get-vaccinated; acquired: 06/12/2021
- Danish Health Authority; “The Danish Health Authority presents plan for revaccination”; https://www.sst.dk/en/English/News/2021/The-Danish-Health-Authority-presents-plan-for-revaccination; acquired: 06/12/2021
- Danish Health Authority; “The Danish Healthy Authority recommends booster vaccination of everyone aged over 18”; https://www.sst.dk/en/English/News/2021/The-Danish-Healthy-Authority--recommends-booster--vaccination-of-everyone-aged--over-18#:~:text=The%20Danish%20Health%20Authority%20recommends,third%20shot%20against%20COVID%2D19.&text=Coronavirus%20infection%20cases%20continue%20to%20rise.; acquired: 06/12/2021
- Danish Health Authority; “In the future, your corona vaccination passport will expire sooner”; https://www.sst.dk/en/English/News/2021/In-the-future_-your-corona-vaccination-passport-will-expire-sooner, acquired: 09/12/2021

ESTONIA

- Ministry of Social Affairs; “The booster dose for people who’re vaccinated with AstraZeneca and Janssen is needed after five months”; https://vaktsineeri.ee/en/news/the-booster-dose-for-people-whore-vaccinated-with-astrazeneca-and-janssen-is-needed-after-five-months/; acquired: 06/12/2021
- Government Communication Unit; “Vaccination in Estonia”; https://kkk.kriis.ee/en/faq/covid-19-vaccination/vaccination-in-estonia; acquired: 06/12/2021

FINLAND

- FINNISH INSTITUTE FOR HEALTH AND WELFARE; "THL has instructed municipalities on giving third doses of coronavirus vaccine to persons vaccinated with short dose interval"; https://thl.fi/en/web/thlfi-en/-/thl-has-instructed-municipalities-on-giving-third-doses-of-coronavirus-vaccine-to-persons-vaccinated-with-short-dose-interval; acquired: 07/12/2021
- FINNISH INSTITUTE FOR HEALTH AND WELFARE; "Getting vaccinated against COVID-19: how, why and when?"; https://thl.fi/en/web/infectious-diseases-and-vaccinations/what-s-new/coronavirus-covid-19-latest-updates/vaccines-and-coronavirus/getting-vaccinated-against-covid-19-how-why-and-when-, acquired: 07/12/2021
- FINNISH INSTITUTE FOR HEALTH AND WELFARE; "Risk groups for severe coronavirus disease"; https://thl.fi/en/web/infectious-diseases-and-vaccinations/what-s-new/coronavirus-covid-19-latest-updates/risk-groups-for-severe-coronavirus-disease; acquired: 07/12/2021

FRANCE

From: Haute Autorité de santé; https://www.has-sante.fr/jcms/p_3165982/fr/coronavirus-covid-19; acquired: from 11/11/2021 to 19/11/2021:

- Stratégie de vaccination contre la Covid-19, Place d'un rappel par le vaccin à ARNm COMIRNATY®, 13 octobre 2021
- Stratégie de vaccination contre la Covid-19, Place du vaccin à ARNm SPIKEVAX, 5 novembre 2021

Other sources available from:

- Ministère des solidarités et de la santé; "La vaccination obligatoire POUR CERTAINES PROFESSIONS", https://solidarites-sante.gouv.fr/IMG/pdf/infog_vaccins_professionnels.pdf; acquired: 07/12/2021
- Ministère des solidarités et de la santé; "La dose de rappel: POUR QUI, QUAND ET OÙ?", https://solidarites-sante.gouv.fr/IMG/pdf/infog_rappel.pdf; acquired: from 03/12/2021 to 07/12/2021
- Service-Public.fr/site officiel de l'administration française; "Passe Sanitaire"; https://www.service-public.fr/particuliers/actualites/A15121; acquired: 02/12/2021

GERMANY

Presse- und Informationsamt der Bundesregierung; "The most important questions and answers about the coronavirus vaccination"; https://www.bundesregierung.de/breg-de/themen/coronavirus/coronavirus-vaccination-faq-1959802; acquired: 14/12/2021

ICELAND

– Directorate of Health; "Reasons for booster vaccinations against COVID-19", https://www.landlaeknir.is/um-embaettid/greinar/grein/item48039/Reasons-for-booster-vaccinations-against-COVID-19; acquired: 15/12/2021

– Directorate of Health; "Booster vaccinations for COVID-19"; https://www.landlaeknir.is/um-embaettid/greinar/grein/item47460/booster-vaccinations-for-covid-19-; acquired: 15/12/2021

– Directorate of Health; "Booster vaccinations for COVID-19 for individuals who have been vaccinated with the Janssen vaccine without a history of previous COVID infection."; https://www.landlaeknir.is/um-embaettid/greinar/grein/item47311/Booster-vaccinations-for-COVID-19-; acquired: 15/12/2021

– Primary Care of the Capital Area; "Vaccination schedule - Week 33"; https://www.heilsugaeslan.is/um-hh/frettasafn/stok-frett/2021/08/11/Vaccination-schedule-Week-33/; acquired: 15/12/2021

– The Directorate of Health and The Department of Civil Protection and Emergency Management; "Booster vaccinations", https://www.covid.is/covid-19-booster-vaccinations; acquired: 15/12/2021

IRELAND

From: Royal College of Physicians of Ireland; https://www.rcpi.ie/policy-and-advocacy/national-immunisation-advisory-committee/ . Acquired: 12/11/2021:

– National Immunisation Advisor Committee (NIAC) INTERIM RECOMMENDATIONS REGARDING BOOSTER DOSES OF COVID-19 VACCINE FOR • THOSE AGED 80 YEARS AND OLDER • THOSE AGED 65 AND OLDER IN LONG TERM CARE FACILITIES; NIAC | 07.09.2021

– National Immunisation Advisory Committee AN OVERVIEW OF RECOMMENDATIONS REGARDING BOOSTER DOSES OF COVID-19 VACCINE FOR HEALTH CARE WORKERS; NIAC | 01.11.2021

– National Immunisation Advisory Committee RECOMMENDATIONS BOOSTER DOSES OF COVID-19 VACCINE FOR • HEALTHCARE WORKERS • THOSE AGED 16-59 YEARS WITH UNDERLYING CONDITIONS • THOSE AGED 50-59 YEARS; NIAC |15.11.2021

– Immunisation Guidelines on COVID-19 Vaccine - Chapter 5a; 05.11.2021

From: https://www.hse.ie/eng/health/immunisation/; acquired: 12/11/2021:

– Clinical Guidance for COVID-19 Vaccination; Version 27; 05/11/2021

Other sources available from:

– Department of Health; Minister for Health announces updates to Ireland's COVID-19 Vaccination Programme - 8 September 2021; https://www.gov.ie/en/press-release/cff1a-minister-for-health-announces-updates-to-irelands-covid-19-vaccination-programme-8-september-2021/#, acquired: 07/12/2021

– Department of Health; Minister for Health announces updates to Ireland's COVID-19 Vaccination Programme - 19 October 2021; https://www.gov.ie/en/press-release/d2092-minister-for-health-announces-updates-to-irelands-covid-19-vaccination-programme-19-october-2021/; acquired: 07/12/2021

– Health Service Executive; "Booster dose of the Covid-19 vaccine"; https://www2.hse.ie/screening-and-vaccinations/covid-19-vaccine/get-the-vaccine/covid-19-vaccine-booster-dose/; acquired: 07/12/2021

ISRAEL

From: Israel's government services and information website; https://www.gov.il/; acquired: 09/12/2021:

– Informed Policies in Covid Protection -Data, Analysis and implications of the third ("booster") Vaccine Israeli MOH, Weizmann Institute of Science, Gertner Institute, Hebrew University & Technion; Oct. 28th, 2021

Other sources available from:

– COVID Information Center, Israel Ministry of Health; "COVID-19 Vaccine for 12-Year-Olds and Older"; https://corona.health.gov.il/en/vaccine-for-covid/over-12/#:~:text=Olds%20and%20Older-,Three%20vaccines%20have%20been%20approved%20for%20use%20in%20Israel%20for,to%20be%20effective%20and%20safe.; acquired: 09/12/2021

– COVID Information Center, Israel Ministry of Health; "Green Pass Guidance"; https://corona.health.gov.il/en/green-pass-lobby/guidelines/; acquired: 09/12/2021

ITALY

From: https://www.salute.gov.it/portale/nuovocoronavirus/archivioNormativaNuovoCoronavirus.jsp?lingua=english; acquired: 03/12/2021:

– CIRCOLARE del Ministero della Salute 27/09/2021; OGGETTO: avvio della somministrazione di dosi "booster" nell'ambito della campagna di vaccinazione anti SARS-CoV-2/COVID-19.

– CIRCOLARE del Ministero della Salute 08/10/2021; OGGETTO: aggiornamento delle indicazioni sulla somministrazione di dosi addizionali e di dosi "booster" nell'ambito della campagna di vaccinazione anti SARS-CoV-2/COVID-19.

– CIRCOLARE del Ministero della Salute 03/11/2021; OGGETTO: aggiornamento indicazioni sulla dose booster dopo vaccinazione con vaccino Janssen.

– CIRCOLARE del Ministero della Salute 04/11/2021; OGGETTO: indicazioni per la dose di richiamo in soggetti vaccinati all'estero con un vaccino non autorizzato da EMA.

– CIRCOLARE del Ministero della Salute 11/11/2021; OGGETTO: Aggiornamento delle indicazioni sulla somministrazione di dosi "booster" nell'ambito della campagna di vaccinazione anti SARS-CoV-2/COVID-19.

– CIRCOLARE del Ministero della Salute 22/11/2021; OGGETTO: Aggiornamento indicazioni su intervallo temporale tra la somministrazione della dose "booster" (di richiamo) e il completamento del ciclo primario nell'ambito della campagna di vaccinazione anti SARS-CoV-2/COVID-19.

– CIRCOLARE del Ministero della Salute 27/11/2021; OGGETTO: estensione della platea vaccinale destinataria della dose "booster" (di richiamo) nell'ambito della campagna di vaccinazione anti SARS-CoV-2/COVID-19.

From: Agenzia Italiana del Farmaco; https://www.aifa.gov.it/; acquired: 03/12/2021:

- Spikevax: raccomandazione EMA sulla dose di richiamo; 25 ottobre 2021, EMA/596529/2021

Other sources available from:

- Ministero della Salute; "Piano vaccini anti Covid 19"; https://www.salute.gov.it/portale/nuovocoronavirus/dettaglioContenutiNuovoCoronavirus.jsp?lingua=italiano&id=5452&area=nuovoCoronavirus&menu=vuoto; acquired: 03/12/2021

- Ministero della Salute, Ministero dell'Economia e delle Finanze e del Ministero per l'Innovazione Tecnologica e la Transizione Digitale; "Certificazione verde COVID-19"; https://www.dgc.gov.it/web/; acquired: 03/12/2021

- Ministero della Saluta; Presidenza del Consiglio dei Ministri; Commissario Straordinario Covid-19; "Vaccino anti COVID-19"; https://prenotazioni.vaccinicovid.gov.it/cit/#/login; acquired: 03/12/2021

LUXEMBOURG

From: https://sante.public.lu/fr/espace-professionnel/recommandations/conseil-maladies-infectieuses.html; Acquired: 19/11/2021:

- Recommandation pour une dose de vaccin à ARN messager d'optimisation après un vaccin COVID-19 Vaccine Janssen; 14 octobre 2021

- Recommandations pour une dose de vaccin SARS-CoV-2 de rappel après une primovaccination; 8 novembre 2021

Other sources available from:

- The Luxembourg Government; Ministry of Health; "Optimisation dose of mRNA vaccine for people who received a single dose of the COVID-19 Vaccine Janssen"; https://gouvernement.lu/en/actualites/toutes_actualites/communiques/2021/10-octobre/18-vaccin-optimisation-janssen.html; acquired: 14/12/2021

- The Luxembourg Government; Ministry of Health; "Additional dose of COVID-19 vaccine after initial vaccination for the elderly and dialysis patients"; https://gouvernement.lu/en/actualites/toutes_actualites/communiques/2021/09-septembre/14-vaccin-dose-additionnelle.html; acquired: 14/12/2021

- The Luxembourg Government; Ministry of Health; COVID-19 vaccination: booster dose after primary vaccination for persons aged 65 years and older and for health professionals; https://gouvernement.lu/en/actualites/toutes_actualites/communiques/2021/11-novembre/09-vaccination-rappel-covid19.html; acquired: 14/12/2021

MALTA

From: Ministry for Health; https://deputyprimeminister.gov.mt/en/health-promotion/covid-19/Pages/mitigation-conditions-and-guidances.aspx; Acquired: 09/12/2021:

- Mandatory Standards and Guidances: Guidance for offices and workspaces; September 2021

Other sources available from:

- Ministry for Health; "Vaccines"; https://deputyprimeminister.gov.mt/en/health-promotion/covid-19/Pages/vaccines.aspx; acquired: 09/12/2021
- Government of Malta, Office of the Deputy Prime Minister; "Malta-Covid-19 vaccination"; https://vaccin.gov.mt/; acquired: 09/12/2021

NETHERLANDS

From: Central Government; https://www.rijksoverheid.nl/documenten/publicaties/2021/11/25/informatieblad-extra-prik-tegen-corona; acquired: 13/12/2021:

- Booster jab against the coronavirus, November 2021

Other sources available from:

- Government of the Netherlands; "Booster vaccination"; https://www.government.nl/topics/coronavirus-covid-19/dutch-vaccination-programme/making-an-appointment-for-vaccination; acquired: 13/12/2021
- National Institute for Public Health and the Environment, Ministry of Health, Welfare and Sport; "COVID-19 booster vaccination"; https://www.rivm.nl/en/covid-19-vaccination/questions-and-background-information/booster-vaccination, acquired: 13/12/2021

Government of the Netherlands; “Coronavirus entry pass”; https://www.government.nl/topics/coronavirus-covid-19/covid-certificate; acquired: 13/12/2021

NEW ZEALAND

From: https://www.health.govt.nz/covid-19-novel-coronavirus/covid-19-vaccines/covid-19-vaccine-information-health-professionals/covid-19-vaccine-policy-statements-and-clinical-guidance; Acquired: from 16/11/2021 to 24/11/2021:

- Booster Vaccination Policy Statement COVID-19, Vaccine and Immunisation Programme, Aotearoa New Zealand, Version 1.0
- AstraZeneca Vaccine Policy Statement Clinical Criteria and Guidance, New Zealand COVID-19 Vaccine and Immunisation Programme, Version 1.2
- Third Primary Dose of the Pfizer/BioNTech Vaccine Policy Statement and Clinical Guidance, New Zealand COVID-19 Vaccine and Immunisation Programme, Version 2.0
- Third Primary Dose of the Pfizer/BioNTech Vaccine Policy Statement and Clinical Guidance, New Zealand COVID-19 Vaccine and Immunisation Programme, Version 3.0

From: https://www.medsafe.govt.nz/; Acquired: 24/11/2021:

- COVID-19 Vaccine AstraZeneca Data Sheet 051121 New Zealand

Other sources available from:

- Ministry of Health – Manatū Hauor; “Mandatory Vaccinations”; https://www.health.govt.nz/covid-19-novel-coronavirus/covid-19-response-planning/covid-19-mandatory-vaccinations; acquired: 07/12/2021
- Ministry of Health – Manatū Hauor; “Booster vaccinations start”; https://www.health.govt.nz/news-media/media-releases/booster-vaccinations-start; acquired: 07/12/2021
- New Zealand Government; “Get your vaccine booster”; https://covid19.govt.nz/covid-19-vaccines/how-to-get-a-covid-19-vaccination/getting-your-booster-dose/#who-is-elegible; acquired: 07/12/2021

NORWAY

- Norwegian Institute of Public Health; "Booster dose for the elderly and nursing home residents"; https://www.fhi.no/en/news/2021/booster-dose-for-the-elderly-and-nursing-home-residents/; acquired: 14/12/2021
- Norwegian Government; "Booster dose for all people over the age of 65 before Christmas and local use of the COVID-19 certificate"; https://www.regjeringen.no/en/aktuelt/booster-dose-for-all-people-over-the-age-of-65-before-christmas-and-local-use-of-the-covid-19-certificate/id2888698/; acquired: 14/12/2021
- Norwegian Government; "Booster dose to be offered to all adults"; https://www.regjeringen.no/en/aktuelt/booster-dose-to-be-offered-to-all-adults/id2886191/; acquired: 14/12/2021
- Norwegian Institute of Public Health; "More people will be offered a booster dose" https://www.fhi.no/en/news/2021/more-people-will-be-offered-a-booster-dose/; acquired: 14/12/2021
- Norwegian Institute of Public Health; "Say yes when you are offered a booster dose" https://www.fhi.no/en/news/2021/say-yes-when-you-are-offered-the-booster-dose/; acquired: 16/12/2021

PORTUGAL

From: https://www.dgs.pt/; Acquired: 12/11/2021:

- Norma no 002/2021 de 30/01/2021 atualizada 08/10/2021; Campanha de Vacinação Contra a COVID-19

Other sources available from:

- SNS; "Third dose of vaccine"; https://www.sns.gov.pt/noticias/2021/10/08/covid-19-terceira-dose-da-vacina/; acquired: 13/12/2021

REPUBLIC OF KOREA

Community Health Center; "Intensive implementation of COVID-19 booster vaccination in nursing homes (11.26)"; http://ncov.mohw.go.kr/en/tcmBoardView.do?brdId=12&brdGubun=125&dataGubun=&ncvContSeq=368835&contSeq=368835&board_id=1365&gubun=; acquired: 09/12/2021

SPAIN

From: https://www.sanidad.gob.es/profesionales/saludPublica/ccayes/alertasActual/nCov/home.htm; Acquired: 22/11/2021:

– Administración de dosis adicionales en personas que han recibido una pauta completa de vacunación frente a COVID-19; Aprobado por Comisión de Salud Pública, 7 septiembre 2021

– Recomendaciones de administración de dosis adicionales y dosis de recuerdo frente a COVID-19 Aprobado por Comisión de Salud Pública, 16 septiembre 2021

– Actualización 9 Estrategia de vacunación frente a COVID-19 en España, Grupo de Trabajo Técnico de Vacunación COVID-19, de la Ponencia de Programa y Registro de Vacunaciones, 18 de octubre de 2021

– Actualización 9 modificada Estrategia de vacunación frente a COVID-19 en España, Grupo de Trabajo Técnico de Vacunación COVID-19, de la Ponencia de Programa y Registro de Vacunaciones, 2 de noviembre de 2021

– Recomendaciones de administración de dosis de recuerdo frente a COVID-19 Aprobado por la Comisión de Salud Pública, el 23 de noviembre de 2021

From: https://facme.es/; acquired: 22/11/2021 :

– Sobre las dosis adicionales de vacunas ARNm frente a la COVID-19; Federación de las Asociaciones Científico Médicas Españolas; Fecha: 14 de octubre de 2021

SWEDEN

- Swedish Civil Contingencies Agency; "Booster shot for more groups"; https://www.krisinformation.se/en/news/2021/september2/booster-shot-for-those-older-than-80-years; acquired: 13/12/2021

- Swedish Civil Contingencies Agency; "People aged between 18 and 65 are recommended to have a third dose"; https://www.krisinformation.se/en/news/2021/november/people-aged-between-18-and-65-are-recommended-to-have-a-third-dose; acquired: 13/12/2021

SWITZERLAND

From: https://www.bag.admin.ch/bag/it/home/krankheiten/ausbrueche-epidemien-pandemien/aktuelle-ausbrueche-epidemien/novel-cov/information-fuer-die-aerzteschaft/dokumente-fuer-gesundheitsfachpersonen.html; acquired: from 18/11/2021 to 10/12/2021 :

- COVID-19 : recommandations relatives à la vaccination de rappel avec un vaccin à ARNm (état au 4.11.2021); Département fédéral de l'intérieur DFI/Office fédéral de la santé publique OFSP/Commission nationale pour les vaccinations CFV

- Recommandations de vaccination avec des vaccins à ARNm contre le COVID-19 (état au 9.11.2021); Office fédéral de la santé publique (OFSP)/Commission fédérale pour les vaccinations (CFV)

- COVID-19 : recommandations relatives à la vaccination de rappel avec un vaccin à ARNm (état au 26.11.2021); Département fédéral de l'intérieur DFI/Office fédéral de la santé publique OFSP/Commission nationale pour les vaccinations CFV

Other sources available from:

- Federal Office of Public Health; "INFORMATION MATERIAL ON THE COVID-19 VACCINATION: FACT SHEETS"; https://foph-coronavirus.ch/downloads/information-material-on-the-covid-19-vaccination-fact-sheets/; acquired: 18/11/2021

UNITED KINGDOM

- Government of the United Kingdom; "JCVI statement, September 2021: COVID-19 booster vaccine programme for winter 2021 to 2022"; https://www.gov.uk/government/publications/jcvi-statement-september-2021-covid-19-booster-vaccine-programme-for-winter-2021-to-2022; acquired: 10/12/2021
- United Kingdom National Health Service; "Immediate action required for Phase 3 booster vaccinations"; https://www.england.nhs.uk/coronavirus/documents/immediate-action-required-for-phase-3-booster-vaccinations/; acquired: 10/12/2021
- Government of the United Kingdom; "JCVI issues advice on COVID-19 booster vaccines for those aged 40 to 49 and second doses for 16 to 17 year olds"; https://www.gov.uk/government/news/jcvi-issues-advice-on-covid-19-booster-vaccines-for-those-aged-40-to-49-and-second-doses-for-16-to-17-year-olds; acquired: 10/12/2021
- United Kingdom National Health Service; "How to get a booster dose of the coronavirus (COVID-19) vaccine"; https://www.nhs.uk/conditions/coronavirus-covid-19/coronavirus-vaccination/how-to-get-a-coronavirus-vaccine/how-to-get-a-booster-dose/; acquired: 10/12/2021

UNITED STATES OF AMERICA

- Centres for Disease Control and Prevention; "CDC Statement on ACIP Booster Recommendations"; https://www.cdc.gov/media/releases/2021/p0924-booster-recommendations-.html#:~:text=Walensky%2C%20M.D.%2C%20M.P.H.%2C%20endorsed,risk%20occupational%20and%20institutional%20settings; acquired: 09/12/2021
- Centres for Disease Control and Prevention; "The Advisory Committee on Immunization Practices' Interim Recommendations for Additional Primary and Booster Doses of COVID-19 Vaccines — United States, 2021"; https://www.cdc.gov/mmwr/volumes/70/wr/mm7044e2.htm; acquired: 09/12/2021
- U.S. Department of Health & Human Services; "Statement from HHS Secretary Xavier Becerra: All Adults Are Now Eligible for COVID-19 Booster Shots"; https://www.hhs.gov/about/news/2021/11/19/statement-hhs-secretary-xavier-becerra-all-adults-are-now-eligible-for-covid-19-booster-shots.html; acquired: 09/12/2021

- Centres for Disease Control and Prevention; "COVID-19 Vaccines for People who are Moderately or Severely Immunocompromised"; https://www.cdc.gov/coronavirus/2019-ncov/vaccines/recommendations/immuno.html#:~:text=PRIMARY%20SERIES%20COVID%2D19%20VACCINE,-Pfizer%2DBioNTech&text=%E2%80%A0-,People%20ages%2012%20years%20and%20older%20who%20are%20moderately%20or,BioNTech%20COVID%2D19%20vaccine%20booster; acquired: 09/12/2021

- Centres for Disease Control and Prevention; "How Do I Find a COVID-19 Vaccine or Booster?"; https://www.cdc.gov/coronavirus/2019-ncov/vaccines/How-Do-I-Get-a-COVID-19-Vaccine.html#:~:text=Find%20a%20COVID%2D19%20vaccine%20or%20booster%3A%20Search%20vaccines.,find%20vaccination%20providers%20near%20you; acquired: 09/12/2021

- U.S. Department of Health & Human Services; "Email Template About COVID-19 Vaccine Boosters"; https://wecandothis.hhs.gov/resource/email-template-about-covid-19-vaccine-boosters; acquired: 09/12/2021

- Centres for Disease Control and Prevention; "COVID-19 Vaccine Boosters"; https://www.cdc.gov/coronavirus/2019-ncov/vaccines/booster-shot.html; acquired: 09/12/2021
